# Supplementary material for: Are carbon emissions trading and green financial instruments synergistic? -Comprehensive quantitative research based on content analysis
Source: PLoS One. 2024 Mar 7;19(3):e0298601. doi: 10.1371/journal.pone.0298601 (PMC10919601; doi:10.1371/journal.pone.0298601)
Supplement: S1 Data — (ZIP) [file pone.0298601.s001.zip › Data/地级市Z_F工作报告.zip!/╜¡╦╒/╠⌐╓▌╩╨/└┤╫╘╣1⁄2╓┌║┼í╛┬φ┐╦╩2╛▌═°í┐_2011.docx]

各位代表：

　　现在，我代表市人民政府向大会作工作报告，请予审议，并请市政协委员和其他列席人员提出意见。

　　一、2010年工作和“十一五”发展回顾

　　过去的一年，在中共泰州市委的正确领导下，我们积极应对宏观形势的新变化，始终坚持以科学发展观总揽全局，紧紧围绕“三年再来一个大变化”目标，大力实施开放创新“双轮驱动”战略，协调推进全市经济、政治、文化与社会建设，圆满完成市三届人大三次会议确定的目标任务。

　　经济平稳较快增长。认真落实促进经济增长的关键举措，经济回升向好势头得到巩固和发展。全年预计实现地区生产总值2000亿元左右，可比增长14%，人均地区生产总值突破6000美元。三次产业增加值分别为148亿元、1127亿元和725亿元，分别增长4.5%、14%和 14.2%。全市财政总收入456.4亿元，增长32.7%，地方一般预算收入170.8亿元，增长23.2%。全社会固定资产投资1538亿元，增长31.9%。社会消费品零售总额550.3亿元，增长19%。农业生产持续发展。深入推进“5218工程”，新增高效农业面积29.6万亩，粮食生产实现“七连增”。新增市级以上农业龙头企业12家、三大合作组织400多家，农业利用“三资”45亿元。新增国家级农业标准化示范区2个，新获认证无公害农产品、绿色食品和有机食品350个。全市森林覆盖率达18%。兴化市再获“全国粮食生产先进县标兵”称号，市农业开发区创成省级现代农业产业园区。成功举办首届农艺节。工业运行质态良好。预计全市规模以上工业销售收入4640亿元、利税550亿元、利润330亿元，分别增长33%、32%和33%。装备制造业、三大新兴产业分别完成产值2050亿元、600亿元，分别增长32%、30%。预计全年工业技改财务发生数950亿元，实施亿元以上重大技改项目192个，其中10亿元以上项目13个。18个项目列入国家、省重点产业振兴和技术改造投资计划。民营经济发展提速，新办私营企业10568家，净增私营个体经济注册资本337亿元，增长71.3%;认真办好中小企业“六件实事”，7家企业跻身中国民营企业500强。实施品牌发展战略，新增中国驰名商标5件。强力推进节能减排，单位地区生产总值能耗完成省定指标，二氧化硫排放量和化学需氧量分别削减27.8%、2%。建筑业加快发展，完成总产值1271.5亿元，增长16.1%，新增一级施工总承包资质企业2家。服务业发展成效明显。深入推进“833工程”，8大服务业集聚区实现营业收入340亿元，30个重点项目完成投资97亿元，30强企业实现利税11.6亿元。金融业发展加快，浦发银行、招商银行、江苏长江商业银行泰州分行开业，兴业银行泰州分行获准筹建，新增农村小额贷款公司22家，“富民阳光信贷”试点取得积极成效。年末全市本外币存款余额2359.8亿元，贷款余额1531亿元，分别比年初增长23.1%和27.1%。亚星锚链、新扬子造船成功上市。旅游经济成为新的亮点，全年接待游客1100万人次，预计实现总收入118亿元。

　　开放创新成效突出。深入实施“双轮驱动”战略，着力提升开放水平、增强创新活力。突出台资、央企招引，精心组织招商活动，建立重大项目绿色通道，总投资80亿元的中海油、9800万美元的扬子江物流等项目成功落户。全年注册协议外资39.7亿美元、实际利用外资13.6亿美元，分别增长46.3%、29%。新批3000万美元以上项目21个。完成自营出口58.8亿美元，实现外经营业额5.8亿美元。全面推进中国医药城各大功能区建设，新开工面积超过100万平方米。正式启动部省共建机制，成功举办苏台生物医药健康产业峰会、首届中国(泰州)国际医药博览会。国家级出口加工区一期封关运作，纬创一期等重大项目即将竣工投产。新能源产业园加快建设，项目集聚势头日益显现。沿江开发纵深推进，在建总投资1亿美元或10亿元人民币以上重大产业项目20个，核心港区建设取得积极进展。口岸工作有序开展，海关、国检、海事、边检为开放型经济发展作出新贡献。外事、侨务和港澳台事务工作取得新成绩。我市再次被台湾电电公会评为值得推荐城市。着力推动创新型经济发展。全年实现高新技术产业产值1552.1亿元，占规模以上工业比重提高1.4个百分点。深化政产学研合作，“企业院校行”成功举办，中科院泰州技术市场网等载体功能明显增强。建成国家级博士后工作站3家、省级工程技术研究中心39家;新增产学研联合体110个、国家重点新产品13个、省级高新技术产品199个、省级创新型企业45家，3家企业被认定为省重点软件企业。入选国家科技重大专项6个、省成果转化资金项目15个，入选数均居全省前列。新获授权专利4198件，建成国家级知识产权维权援助中心。全年引进创新创业人才团队54个、高层次人才805名，其中海外优秀人才167名。教育部“蓝火计划”在高港启动。各市(区)均创成全国科普示范县(市、区)。

　　城乡建设扎实推进。坚持市区一体，强化资金筹措，推行阳光拆迁，加快建设进度，市区城建投资超过150亿元。新一轮城市总体规划修编通过部省联合审查，城乡规划体系逐步健全。强力推进“十大工程”建设。中国医药城会展交易中心一期投入使用，规划展示馆建成，博物馆、图书馆、美术馆主体封顶，华侨城一期、三水湾一期开业，凤城河环河整治、城市水生态环境建设、老通扬运河整治二期、青少年科技活动中心和职工活动中心、周山河街区“四路七桥”、高港大道等工程加快实施，人民医院新院、新区高级中学前期工作有序开展。万达城市综合体、省泰中老校区改造、稻河古街区民俗文化展示中心加快建设，莲花学校二期、革命烈士纪念馆一期竣工。迎春路东延即将贯通，京泰客运站主体完工，火车站街区启动建设。深入推进文明城市创建，着力提升城市管理水平，创成省优秀管理城市。各市加大城建力度，靖江滨江新城核心区建设成效明显，泰兴“三城四片五重点”城乡建设格局初步形成，姜堰创建国家卫生城市通过国家技术评估，兴化城市“水文特色”进一步彰显。继续加强新农村建设，全市新(改)建农村公路500公里，改造农村桥梁200座，改厕91435座，疏浚河道3019万方;继续实施全面小康村“十百千”工程，新创市级先行村300个;大力推进“125工程”，重点镇村规划编制基本完成，项目建设不断加快。推进重大基础设施建设，泰州长江大桥及北接线工程加快实施，江海高速、328国道改线建成通车，长江大道先导段基本建成。4条省道新建或改线、周山河船闸改造、卤汀河拓浚等工程开工建设。电力、邮政、通信等基础设施建设步伐加快。

　　社会事业加快发展。推进区域教育现代化创建，靖江、泰兴、高港通过省级验收;促进义务教育均衡发展，24所“公有民营”学校回归公办;加快发展高等教育，南京中医药大学翰林学院建成招生。加强文化泰州建设。历史文化名城保护规划获批，国家历史文化名城申报通过专家评估。完成北山寺改造、岳庙修复和周氏住宅修缮主体工程，兴化金东门历史文化街区对外开放。泰州淮剧和茅山号子入选国家级非物质文化遗产名录，3人入选省非物质文化遗产传承人。歌舞《荡湖船》获全国群星奖，9部戏剧作品获全国戏剧文化奖。加快乡镇(街道)宣传文化中心和村文化室建设，农家书屋实现全覆盖。泰州报业传媒集团和广播电视传媒集团成立，文化旅游发展有限公司组建。成功举办国际旅游节、梅兰芳艺术节，承办省新人新作歌舞大赛。调整优化卫生资源配置，基本药物制度加快实施，公立医院改革试点取得新进展。积极组织参加省第十七届运动会，奖牌总数排名比上届前移5位，荣获青少年部进步奖全省第一名。人口出生率稳定在较低水平，对持“独生子女光荣证”的市区企业退休职工发放一次性奖励，市计生协会被表彰为“全国先进集体”。认真抓好第六次全国人口普查工作。深入实施生态市建设行动计划，加快推进城镇污水处理设施建设，企业违法排污整治、秸秆烟害防控和能源化利用等工作取得新成效。加强耕地保护，土地集约利用水平进一步提高。市民防局荣获“全国人民防空先进单位”。市供销社成为全国总社改革发展联系点。史志档案、社会科学、无线电管理、气象、防震和民族宗教、妇女儿童工作继续加强，质监、银监、工商、税务管理为地方经济发展作出新贡献，国防教育、国防动员、民兵预备役、双拥工作取得新成绩。

　　人民生活明显改善。落实防通胀、稳物价等政策措施，办成一批改善民生实事，着力提升人民群众生活水平。预计全市城镇居民人均可支配收入20250元，农民人均纯收入9240元，分别增长12%、13%。以创建国家级创业型城市为抓手，认真实施创业带动就业行动计划，全年净增城镇就业6万人、失业人员再就业2.9万人，新增创业5.5万人。推进城乡居民养老保险全覆盖，参保城镇职工和居民医药费报销比例及支付限额大幅提高。新型农村合作医疗参保率达99.6%，政府补助4.2亿元，受益农民320万人。工伤保险实现市级统筹“五统一”。城乡低保实现动态管理下的应保尽保，标准分别提高到每月340元和210元。市区残疾人养老保险全面启动，向4556名重度残疾人发放护理补贴。医疗救助专项资金累计支出2353.5万元，救助11.3万人次。初步建立普惠型社会福利体系，全市新增居家养老服务站348家、省级示范性居家养老服务中心4家，五保集中供养率达65%。完善抚恤补助标准自然增长机制，共向21905人发放补助金1.3亿元。慈善事业长足发展，救助对象近6万人。加强住房保障工作，全市新增保障性住房9505套。市区公交发展有了根本性改善，公交车万人拥有量比上年增加1倍，新建首末站5个，新辟、优化线路26条。深化放心消费创建，市区5家农贸市场完成升级改造。区域供水工程泰兴、姜堰主管网建成，农村饮用水安全工程新增受益人口47.7万人。定期举办“百姓大学堂”等市民素质教育活动，市“百姓名嘴”宣讲队荣获全国基层理论宣讲先进集体。扎实抓好基层基础工作，全市安全生产形势总体平稳。加强食品药品安全监管，保障人民生命健康安全。

　　自身建设继续加强。加大对经济工作的组织推进力度，制定出台《实施开放创新双轮驱动战略，推进经济转型升级的若干政策》、《加快市区民营中小企业发展政策意见》、《加快市区现代服务业重点产业发展的奖励办法》等政策意见。深化行政管理体制改革，政府机构改革顺利推进，医药高新区组建。“网上政府”建设取得新成效，行政权力网上公开透明运行通过省考核验收。积极推行新闻发言人制度，认真办好市长公开电话、市长信箱和行风热线。自觉接受市人大及其常委会的监督和市政协的民主监督，注重发挥民主党派、工商联、无党派人士和工会、共青团、妇联等群众团体作用，认真办理人大代表建议和政协提案，满意(含基本满意)率100%。加强平安泰州、法治泰州建设，严格落实信访维稳工作责任制，积极推进干部大走访，不断完善大调解机制，保持社会稳定。强化应急管理，妥善处置各类突发事件，江苏省(泰州)应急救援中心一期建成启用。圆满完成对口援建四川绵竹拱星镇各项任务，认真做好对口支援新疆昭苏县工作。加强基层民主法制建设，“五五”普法成效明显，村民委员会、社区居委会换届选举顺利完成。开展村组(社区)股份合作制改革，市区114个村、14个组完成改革，80个村撤村建居。严格执行党风廉政建设责任制和《廉政准则》，源头治腐和纠风治乱力度加大;强化效能监察和监督检查，执行力得到新提升。机关公有资产管理进一步加强，节约型机关建设取得积极进展。

　　在充分肯定成绩的同时，我们也清醒地看到，全市经济社会发展中还存在一些问题和不足。主要是：经济结构性矛盾仍然突出，节能减排压力较大;高层次人才等资源要素缺乏，核心竞争力不够强;中心城市功能尚不完善，城乡统筹发展还需不断强化;社会保障水平需要进一步提高，就业形势不容乐观;城乡居民持续增收难度加大，困难群体生活有待进一步改善;政府自身建设还存在一些薄弱环节，行政管理体制和行政效能滞后于发展要求，社会建设和管理能力需要加快提升，等等。对上述问题，我们将采取切实有效措施，在今后的工作中努力加以解决。

　　各位代表，刚刚过去的2010年是实施“十一五”规划的最后一年。五年来，我们始终坚持科学发展，既重视打牢经济基础，又重视彰显特色和优势，更重视改善民生、促进和谐，全市经济社会发展取得巨大成就，既定的主要发展目标圆满完成。

　　坚持加快发展，优化经济结构，综合实力明显提升。预计2010年全市地区生产总值是“十五”期末的2.3倍，年均增长14.4%;财政总收入、地方一般预算收入分别是“十五”期末的4倍、3.6倍，年均分别增长32%和29.3%;累计完成全社会固定资产投资4841亿元，是“十五”的3.8倍，年均增长30.6%;本外币存贷款余额分别是“十五”期末的2.8倍、3.4倍。高效农业、新兴产业和现代服务业成为三次产业发展的重要支撑，三次产业结构由“十五”期末的11.1:57.8:31.1调整为7.4:56.4:36.2，“1+3+N”产业发展格局初步形成。建成全国唯一的国家级医药高新区，成为“十一五”发展最突出的亮点。

　　坚持创新发展，深化改革开放，增长动力持续增强。行政管理、市区体制、社会体制等关键领域改革取得重要进展，区划调整稳步推进，农村税费改革成效明显，投融资体制改革不断深化。累计实际利用外资51.1亿美元、自营出口201.4亿美元，分别是“十五”的3.4倍、5.4倍。国家医药高新区和出口加工区的建成，实现了招引重大产业项目和重大载体建设的历史性突破。私营个体经济注册资本1337.5亿元，是“十五”期末的4.1倍。高新技术产业产值占规模以上工业比重比“十五”期末提高4.1个百分点。连续三次被评为全国科技进步先进市，建成全国知识产权工作示范市。

　　坚持统筹发展，改善人居环境，城乡面貌显著变化。中心城市建设累计投入520亿元，是“十五”的2.5倍。城市框架进一步拉开，北部老城区改造、南部新城区建设、滨江港城区发展全面加快，建成区面积扩大到61平方公里。创成国家卫生城市、国家环保模范城市、国家园林城市、中国优秀旅游城市和全国双拥模范城市。城乡统筹发展步伐加快。中小城市、重点镇村梯次推进、联动发展的格局初步形成，城市化水平达53%。新农村建设取得积极进展，农村新“5+1”实事顺利完成。泰州电厂、苏中机场、泰州长江大桥、宁启铁路复线电气化改造、江海高速等一批重大基础设施项目相继实施建设，有的已建成投运。区域经济竞相发展，市区经济发展加快，四市均进入全国百强县行列。

　　坚持协调发展，强化公共服务，社会事业全面进步。全面实现城乡义务教育免费入学，教育教学质量稳步提升;职业教育服务功能不断增强，高等教育规模和层次实现重大跨越。医药卫生体制改革扎实推进，基层医疗卫生服务体系基本建成。加强文化泰州建设，推动文化体制改革，积极发展文化产业，着力构建公共文化服务体系。妇女儿童、人口计生、民政福利、社会救助等社会事业发展取得新进展。环境质量综合指数稳定在85以上，四市全部创成国家级生态示范区。

　　坚持和谐发展，着力富民安民，人民生活更加殷实。以县为单位，全部达到省定小康目标，全市总体实现全面小康。城乡居民收入分别是“十五”期末的1.9倍和1.8倍，年均分别增长14.2%和12.6%。就业服务体系建设扎实推进，累计新增城镇就业33.7万人，登记失业率始终控制在3.5%以内。在全省率先构建多层次全覆盖的城乡社会保障体系，三年脱贫攻坚任务全面完成，住房体系初步形成，人民群众生活质量和环境有了新的改善。创新信访工作机制，在全国率先建立党政干部下访制度，成为全省社会治安综合治理先进市。

　　“十一五”经济社会发展的巨大成就，为“十二五”发展夯实了基础、积蓄了力量。这些成绩的取得，是全市人民在中共泰州市委领导下，团结一致、开拓创新、克难奋进的结果，与市人大、市政协的监督、支持密不可分。在此，我代表市人民政府，向辛勤工作在全市各行各业的广大干部群众，向给予政府工作热情支持和有力监督的全体人大代表、政协委员，向各民主党派、工商联、无党派人士、各人民团体和广大离退休老同志，向中央和省驻泰机构以及驻泰部队指战员、武警官兵和公安干警，向所有关心支持泰州发展的海内外朋友们，表示衷心的感谢!

　　二、“十二五”发展目标任务

　　各位代表，“十二五”是我市全面建设更高水平小康社会、向率先基本实现现代化迈进的重要时期，是加快转变经济发展方式、推动经济社会转型升级的关键阶段。为谋划未来五年发展，2009年以来，市政府组织开展了“十二五”规划编制工作。党的十七届五中全会以后，按照中央和省委的要求，根据市委三届十次全会通过的《中共泰州市委关于制定泰州市国民经济和社会发展第十二个五年规划的建议》，市政府制定了《泰州市国民经济和社会发展第十二个五年规划纲要(草案)》，提请大会审议。

　　“十二五”时期，经济社会发展的指导思想是：坚持以邓小平理论和“三个代表”重要思想为指导，深入贯彻落实科学发展观，以“推进富民强市、建设美好泰州”为主题，以加快转变经济发展方式为主线，以开放创新“双轮驱动”为动力，以新型工业化和新型城镇化为导向，着力推进经济结构调整，着力统筹城乡发展，着力加强文化泰州建设，着力保护生态环境，着力促进社会和谐稳定，努力走出一条具有泰州特色和优势的发展新路，向率先基本实现现代化迈进。

　　“十二五”时期，经济社会发展的主要目标是：地区生产总值年均可比增长12%左右，到2015年达4000亿元左右，比2010年翻一番，人均地区生产总值达85000元左右。财政总收入、地方一般预算收入年均增长15%。累计实际利用外资100亿美元，外贸进出口总额220亿美元。全社会固定资产投资、社会消费品零售总额年均增长16%。城镇居民人均可支配收入、农民人均纯收入年均增长12%左右，低收入者收入明显增加。

　　“十二五”发展的重点任务：一是构建现代产业体系，提高产业竞争力。坚定不移地走新型工业化道路，着力打造“1+3+N”产业体系。积极优化产业布局，加快构筑“一城一区一园一带一块”产业空间格局。坚持现代服务业与先进制造业融合互动，推动服务业发展提速、结构提升、层次提高，服务业增加值年均增长13%，生产性服务业比重逐步提高。积极发展现代农业，高效农业比重稳步提升。二是实施“双轮驱动”战略，建设开放创新城市。举全市之力建设中国医药城，基本建成“中国第一、世界有名”的医药高新区。坚持高起点引进、高位嫁接和高水平产学研合作，加强创新能力建设和产业创新支撑。研发经费支出占地区生产总值比重达2.5%，高新技术产业产值占规模以上工业比重达38%以上。更加注重招引新兴产业、优势主导产业重大项目，利用外资质量、规模不断提升。大力实施人才强市战略，坚持人才优先发展，加快建设人才高地。依托城市和开发园区，优化开放创新环境。三是突出中心城市建设，提升集聚辐射能力。积极改造老城区，加快建设新城区，大力发展港城区，进一步完善城市发展格局。健全城市社会服务、商贸服务、公共交通、公用设施四大体系，不断完善城市功能。精心打造生态宜居城市，着力提升城市品质。优化产业布局，打造“五大板块”，做强市区经济。四是统筹区域协调发展，加快城乡一体化步伐。坚持走新型城镇化道路，加快建设新农村，着力构筑中心城市、副中心城市、小城市、特色城镇协调发展的格局。更高层次统筹区域发展，加快形成中心城市、沿江地区、里下河地区及黄桥老区等三大经济区协调发展的新局面。加强城乡重大基础设施建设，为经济社会发展提供有力支撑。城市化水平达60%左右。五是加强社会建设与管理，着力保障和改善民生。坚持经济增长与就业增长良性互动，促进社会充分就业，五年累计新增城镇就业30万人。完善社会保障体系，城乡居民社会保障基本实现全覆盖，社会保障水平进一步提高。坚持政府保障与市场供给相结合，不断完善保障性住房体系和商品房体系，防止房价过快上涨，促进房地产市场平稳健康发展。全面发展各项社会事业，继续推进教育现代化，泰州大学筹建取得实质性进展，建成教育强市;加强公共卫生服务体系建设，建成覆盖城乡的基本医疗卫生制度，着力构建多元办医格局。加强民主法制建设，推动社会管理创新，保持社会和谐稳定，人民群众对社会治安满意率保持98%以上。六是推进文化泰州建设，全面提升文化软实力。扎实推进文明城市创建，努力创成全国文明城市。加快发展文化事业，力争跨入国家历史文化名城行列。大力发展文化产业，积极推进文化创新，努力形成富有活力、形态多样的文化产业集群。七是提高生态文明水平，促进可持续发展。加强生态建设和保护，持续改善环境质量，创成国家生态市。加快建设资源节约型社会，努力建成国家级节水型城市。单位地区生产总值能耗下降率、二氧化碳排放削减率、主要污染物排放削减率达到省定目标要求，环境质量综合指数保持在85以上，森林覆盖率达22%左右。八是加快改革步伐，激发体制机制活力。着力转变政府职能，创新公共服务方式，实现基本公共服务提供主体和提供方式的多元化，促进非基本公共服务市场化。完善市区管理体制，推动一般性、具体性工作任务重心下移，实现重大规划、重大项目的统一协调。深化投资体制改革，强化企业投资主体地位，提高政府投资决策水平。大力发展民营经济，扩大民间资本投资领域。创新农村发展机制，积极推进农村综合改革。

　　三、2011年工作任务

　　各位代表，今年是建党90周年、建市15周年，是实施“十二五”规划的开局之年，也是经济社会发展“三年再来一个大变化”的成果展现之年，做好今年的工作具有十分重要的意义。我们将全面贯彻党的十七届五中全会、省委十一届九次全会精神，认真落实市委三届十次全会决策部署，突出主题、贯穿主线，激发动力、明确导向，全面提升经济社会发展水平。

　　全市国民经济和社会发展主要调控目标为：地区生产总值增长12%;财政总收入增长15%，一般预算收入增长15%;社会消费品零售总额增长18%;全社会固定资产投资增长20%;实际利用外资12亿美元;外贸进出口总额增长15%以上;城镇居民人均可支配收入增长12%，农民人均纯收入增长12%;居民消费价格总水平涨幅控制在4%左右;城镇登记失业率控制在3.5%以内;研发经费支出占地区生产总值比重1.9%，高新技术产业产值占规模以上工业比重32%以上;单位地区生产总值能耗下降率、二氧化碳排放削减率、主要污染物排放削减率达到省定目标要求。

　　今年将重点抓好五个方面的工作：

　　1、加快转变经济发展方式，大力推进新型工业化。认真落实“双轮驱动”政策意见，突出产业结构优化升级，着力提升优势特色产业发展规模和层次。

　　打造先进产业体系。实施“传统优势产业升级计划”。积极运用高新技术和先进适用技术，促进以船舶为重点的装备制造业抢占产业制高点，推动石油化工、精细化工特别是高分子复合材料等特色化工的发展，提升家电、冶金、纺织等其他传统产业的技术、品牌优势，力争传统产业销售收入达4500亿元。实施“新兴产业双倍增计划”。加快生物技术和新医药产业发展，着力构建完整产业体系，医药高新区力争创成“国家级新型工业化示范基地”;充分发挥纬创项目的带动示范效应，加快集聚配套企业和研发机构，做大做强电子信息产业;主攻太阳能光伏和动力电池，加快产业集聚和产业链延伸，壮大新能源产业规模;积极培育新材料、智能电网、节能环保装备等若干个新兴产品集群。力争新兴产业销售收入达750亿元。实施“规模企业培育计划”，支持和鼓励规模企业上台阶、中小企业上规模，重点实施200个亿元以上重大技改项目，技改财务发生数增长20%以上，新增销售收入超百亿元的企业1家。实施“服务业提速计划”。加速发展生产性服务业，提升发展生活性服务业，积极培育新兴服务业，突出抓好六大产业发展和重点集聚区建设，新增百亿级服务业集聚区、省百强服务业企业各1家。

　　强化产业创新支撑。围绕创建国家创新型试点城市，积极推动高层次人才队伍建设、高新技术产业发展、高科技成果转化。深入实施“凤城千人计划”，着力优化培养、引进、集聚、用好高层次人才的政策、机制和环境，加快建设人才强市。力争引进创新人才团队60个、高层次人才800名，其中海外优秀人才200名。加快发展高新技术产业，大力培育创新型企业。新增省级以上创新型企业20家、市级以上高新技术企业50家、高新技术产品120个。推进“千企科技服务工程”，培育重大自主创新项目20项;加强以企业为主体的创新体系建设，新增省级院士工作站7个，新建产学研联合体100个，新增中国驰名商标3件。大力推进资本运作，扎实抓好百企上市培育，积极筹建科技银行，鼓励发展风险投资、创业投资。

　　推进节能减排工作。加强源头控制、科技支撑、严格监管，进一步降低能源资源消耗、减少污染排放。大力淘汰落后产能，严控产能过剩行业新上扩能项目。积极发展绿色经济、循环经济和低碳技术，推进重点节能减排工程建设。认真落实监管措施，严格执行节能评估审查制度和环境准入标准。加大环境保护力度，扎实开展国家生态市创建。年内，确保通过国家环保模范城市省级复查验收，建成全国节水型社会建设示范市。

　　统筹区域经济发展。引导各地立足产业基础和比较优势，做大做强主导产业和特色产业，不断提升综合竞争力。优化沿江地区产业布局，拓展开发空间，推进跨江联动，加快融入苏南经济板块。大力发展市区新兴产业和现代服务业，加快建设产业高地和区域性服务中心。认真落实各项政策措施，推动里下河地区和黄桥老区加快发展。

　　2、深化改革开放，进一步增强经济发展活力。坚持以改革促开放、以开放促发展，始终把改革开放作为增强发展活力、推动跨越发展的强大引擎。

　　进一步提高对外开放质量和水平。围绕新兴产业、特色产业，大力引进实施重大投资项目和拥有自主知识产权的项目。探索股权私募基金等利用外资新形式，鼓励现有外资企业增资扩股。突出高端项目和产业链招商，建立高层次招商信息网络。办好第二届中国(泰州)国际医药博览会及科技、经贸洽谈会等重大活动，力争在重大产业项目招引上取得新突破。全年力争引进超3000万美元项目25个，其中过亿美元项目2个。加快转变外贸发展方式，优化对外贸易结构，提高一般贸易、服务贸易、自主知识产权和自主品牌产品出口比重，力争年出口超3000万美元企业20家以上。加强出口品牌、基地培育，积极创建国家船舶出口基地。鼓励企业在更高层次上利用“两种资源”、“两个市场”，切实加快“走出去”步伐。加强外事、侨务工作，促进对外交流合作。

　　着力构建产业开放格局。发挥“一城一区一园一带一块”主体作用，为对外开放提供更好投资平台和更大发展空间。坚定不移地举全市之力建设中国医药城，加快产业化、资本化进程。吸纳一批国内外最新医药创新成果落地生产，推动3-4家世界前十强医药企业落户园区，引进一批年销售10亿元以上的医药产品，培植一批年销售10亿元以上的企业，建成100条符合GMP规范的生产线;筹建生物医药产业基金，推动中国医药城(集团)股份有限公司早日上市。加快出口加工区二期建设，建立符合国际惯例、高效运转的管理体制和运行机制。高起点、高标准、高规格推进新能源产业园规划建设，力争创成省新能源产业基地。深化沿江开发，推进核心港区建设，全力打造以临港产业为重点、以现代物流业为主体的沿江产业带。坚持着眼高端，提升内涵，彰显优势，着力打造里下河特色经济板块。引导、支持各省级开发区和特色园区配套完善公共平台，切实增强产业吸纳能力。

　　深化重点领域改革。着手实施各市政府驻地行政管理制度改革，切实抓好街道、社区建设工作。全面解决市区城中村、城郊结合部行政管理体制问题，加强新老社区建设，进一步提升基层基础工作水平。继续完善市区行政、财政、城建体制，激发市区发展活力。认真抓好国家小城镇发展改革试点、国家和省经济发达乡镇行政管理体制改革试点和农村产权改革试点工作。深化职业教育办学体制改革、文化管理体制改革，深入推进公立医院改革试点。

　　大力发展民营经济。深入实施《民营经济三年跃升计划》，全年净增私营个体经济注册资本200亿元、新办私营企业7000家。引导民间资本投资新兴产业，支持中小企业“二次创业”，鼓励民营企业集聚特色园区。加大信贷支持力度，推广“富民阳光信贷”，加快银行机构向县域延伸，鼓励新办小额贷款公司、村镇银行。推进“一中心五平台”服务体系建设，加强民营企业家队伍建设，加快建立现代企业制度，促进民营经济更好更大发展。

　　3、促进农业增效、农民增收，扎实抓好“三农”工作。坚持城乡统筹发展，加大强农惠农力度，夯实农业农村发展基础。

　　大力发展现代高效农业。继续实施“5218”工程，加强农业园区和特色基地建设，建成10个万亩现代农业园区，力争创成1个国家级现代农业示范区，新增高效农业面积20万亩。加快实施万顷良田建设工程，深入开展粮食高产增效示范创建，新增粮棉油万亩示范片30个。兴化市继续保持粮食总产、单产全省第一。推进农业产业化、市场化进程，市级以上龙头企业销售收入、利税分别增长20%、15%，农业利用“三资”力争40亿元以上。重点推广30项农业新品种、新技术、新模式。加强农产品品牌建设，促进农产品提质升档。切实抓好林业绿化工作，力争全市森林覆盖率达20%。强化农产品质量安全管理，保障放心食品供应。

　　继续抓好新农村建设。认真办好新一轮实事，着力改善农村生产生活条件。加强农田水利建设，加快大中型灌区改造，完善小型水利设施。推进农村交通建设，新(改)建农村公路300公里，改造桥梁250座。抓好村庄环境综合整治，加快秸秆能源化利用，整治疏浚农村河道，完善垃圾收运处理体系。深入推进农村饮用水安全工程，新增受益人口42万人。实施全面小康村“十百千”提升工程，创成达标村和示范村各100个。大力发展村级集体经济，加大对经济薄弱村帮扶力度，重视做好债务化解工作。

　　努力促进农民增收。大力发展订单农业，建立健全紧密合理的企业、农户利益联结机制，提升农户种养效益。推进农业适度规模经营，引导开展农村土地流转，新增规模经营面积15.4万亩。完善农业生产经营机制，年内新增农村三大合作组织300家以上。强化农民就业创业培训，着力培养新生代农民。继续抓好全国供销总社改革发展联系点各项工作，推动供销系统改革发展，进一步提升为农服务水平。

　　加快新型城镇化进程。扎实推进“125工程”，深入开展重点镇村建设。进一步完善规划设计，合理确定功能定位，着力体现个性特征，加快推动城镇建设的特色化。更加注重城镇经济发展，加快推动产业就业结构的多样化。打造宜居环境，加快推动城镇生态化转型。积极创造条件，为外来人员务工、农村劳动力转移提供平等公共服务，加快推动市民化进程。

　　4、推进中心城市建设，提升集聚辐射能力。严格执行第三轮城市总体规划，加强城市建设和管理，做美做精中心城市。

　　不断优化空间形态。加强中心城市与周边的规划衔接，推进基础设施统一布局、共建共享，努力促进“同城化”发展。加快外围快速通道建设，继续做好苏中机场和泰州长江大桥建设的服务协调工作，加快宁启铁路复线电气化改造，推进大桥北接线、泰镇高速和231省道泰高段建设，建成长江大道。完善城市路网结构，实施鼓楼路北延、森园路西延等畅通工程，开工建设站前路一期，加快永定东路、南通路二期、海陵中路、海陵北路等道路改造，高港大道主体工程竣工。医药高新区全面实施“路桥连网”工程，高港区拉开“五纵九横”道路框架。

　　加快完善城市功能。着力提升文化旅游功能，推进稻河古街区改造复兴，建成民俗文化展示中心，实施三水湾二期、柳敬亭和南山寺公园、盐税文化广场等工程，开工建设清真寺。着力提升城市商贸功能，启动建设医药高新区中央商务区，加快万达、深茂等城市综合体和泰州国际、世贸雅思等五星级酒店建设，实施东、西大门和青年南路街区改造、迎春路和东进路等街道立面改造，推进滨江新城和火车站街区建设。着力提升公共服务功能，加快新区高级中学、人民医院新院建设，启动建设体育公园，建成青少年科技活动中心和职工活动中心，实施汽车西站迁建，加强停车场所建设，推进污水管网完善工程和城市电网改造，建成气象雷达站。

　　精心打造生态宜居城市。推进凤城河环境综合整治和卤汀河、泰东河拓浚，启动引江河二期;推行河道生态护坡，完成水生态环境封闭控制工程，确保水体水质明显改观。实施凤城河绿化亮化景观工程，建设一批绿色走廊和公共绿地，推进大桥生态公园、海军舰艇主题公园和滨江湿地公园建设。大力提升住宅小区建设品质，加快实施城中村、老小区改造，进一步完善物业管理长效机制。坚持建管并重，加强城市管理，打造“百姓城管”品牌。扎实做好国家卫生城市复查迎检工作，确保通过验收。

　　5、以保障改善民生为出发点和落脚点，加快构建和谐社会。顺应人民群众过上更加美好生活的新期待，创新社会建设和管理，让人民群众共享改革发展成果。

　　突出抓好就业和社会保障工作。坚持创业富民的优先导向，调整优化收入分配结构，着力完善企业职工工资正常增长机制，进一步扩大中等收入群体。深入推进“双全”工程，创成国家级创业型城市。推进城乡统筹就业，继续抓好大学生就业工作，完善面向困难群众的就业援助制度，净增城镇就业4.2万人。推进各类人员参加社会保险，加快实现全覆盖。探索医疗保险市级统筹，继续扩大报销病种和药物范围，提高参保人员待遇。加强城乡低保动态管理，全面落实农村五保政策，完善特困救助制度，实施家庭经济困难学生资助计划。健全被征地农民社会保障机制，加快实现城市规划区内被征地农民保障与城镇职工基本保障接轨。全面开展残疾人养老保险工作，逐步将残疾人康复医疗项目纳入医疗保险范围。重视人口老龄化问题，出台扶持民办养老机构发展政策，新增170家省级社区居家养老服务站，开设3家市区街道老年活动中心。保持房地产市场平稳健康发展，着力解决中低收入家庭住房困难，全市新增保障性住房5770套，市区新开工建设拆迁安置房200万平方米以上。强化重要农副产品市场和价格监管，落实好“米袋子”、“菜篮子”负责制。

　　协调发展社会事业。进一步提高学前教育普及水平，加强农村中心幼儿园建设，全市新增省级优质幼儿园15个以上;大力推进义务教育优质均衡发展，加快普通高中内涵发展;优化发展职业教育，力争创成1个省级职业教育创新发展实验区;加快发展高等教育，继续推进泰州大学筹建和中国医药城教育教学区建设。巩固完善医疗卫生服务网络，加快三级甲等综合医院建设，实施市区社区卫生服务机构建设提升工程，加强县级医院、中心乡镇卫生院和标准化村卫生室建设;全面实施基本药物制度，提高新型农村合作医疗筹资水平和补偿标准。继续优先发展公交，市区新增(更新)公交车150辆，新辟、优化线路14条，建成智能中心，推进城乡客运一体化。完善公共体育设施，加快农村、社区体育工程建设，提升全民健身服务水平。落实计生保障优惠政策，改善对流动人口的服务。继续做好第六次全国人口普查工作。加快发展社会福利和各项慈善事业。深入推进人民防空与防灾一体化建设。认真做好史志档案、社会科学、无线电管理、新闻、统计、气象、防震和民族宗教、妇女儿童等工作。

　　加快建设文化泰州。坚持文化泰州建设与文明城市创建同步推进，城市管理水平和市民文明素质同步提升，进入全国文明城市创建工作先进市行列。用足用好优惠政策，设立文化产业发展专项引导资金，强化企业和项目招引，大力发展文化生态旅游和文化创意产业。推进凤城河风景区5A级景区创建，支持溱湖风景区、华侨城建设和开发，加快筹建泰州文化创意产业园区。加大改革创新力度，激发文化产业发展活力。完善公共文化服务体系，继续加快覆盖城乡的文化设施建设。实施“文化民生”工程，推进城市文化下乡、农村文化进城。完善奖励制度，鼓励文艺创作，推进文艺创新。建立健全文化人才选用和激励机制，着力培养和引进高层次文化人才。做好国家历史文化名城申报后续工作，办好建市15周年、第三届中国泰州国际旅游节、第三届梅兰芳艺术节等重要活动。

　　加强精神文明和民主法制建设。贯彻落实《公民道德建设实施纲要》，创新公民道德实践载体，放大“百姓”、“爱心”系列品牌效应。深化群众性精神文明创建，组织开展行业和市民文明指数测评。深入实施未成年人思想道德建设“三年提升行动计划”，建成未成年人社会实践基地。积极开展双拥共建活动，做好国防教育、国防动员、民兵预备役和兵员征集工作。以创建法治城市为抓手，加快法治泰州建设。坚持“四民主、两公开”，推进农村基层民主法制建设。抓好“六五”普法，加强法律援助工作。

　　切实维护社会稳定。落实信访维稳工作领导责任制，坚持领导干部定期接访和下访，深化大调解机制建设，依法处理好信访突出问题;落实社会稳定风险评估制度，最大限度地从源头上预防和减少社会矛盾。深化平安泰州建设，加强基层社会管理，开展和谐村(社区)示范创建，依法打击各类违法犯罪活动，切实提高人民群众安全感。更加重视应急保障工作，努力提高应急管理水平。开展“安全生产基层基础提升年”活动，始终保持安全监管高压态势，严防重特大事故发生。实施食品、药品放心工程，保障人民群众生命健康安全。

　　四、推进服务型政府建设

　　各位代表，我市经济社会发展已经站在新的历史起点上，“十二五”发展的新任务和人民群众的新期盼对政府工作提出了新的更高的要求。必须进一步转变政府职能，推进服务型政府建设，努力开创政府工作新局面。

　　不断提高统筹能力、创新能力、执行能力。着眼全局、统筹兼顾，突出抓好对全局具有带动作用的重点工作，埋头推进打基础、利长远的重大项目，坚决维护广大人民群众的根本利益和切身利益，更好地把改革的力度、发展的速度和社会可承受的程度统一起来。解放思想、开拓创新，准确把握追赶型城市的阶段性特征，始终保持奋发有为的精神状态，充分激发全社会的创造活力，勇于在开放创新中把“不可能”变成“可能”，把“不现实”变成“现实”。只争朝夕、锲而不舍，坚决把市委的各项决策部署落到实处，坚决把各项目标任务和政策措施不折不扣地落实到位，确保政令畅通、一抓到底。

　　始终坚持科学行政、民主行政、依法行政。把科学决策作为科学行政的首要环节，多渠道发扬民主，广泛听取各方面意见，进一步完善重大事项集体决策、专家咨询、社会公示和听证制度，努力使发展思路、工作部署和政策措施更加贴近实际、顺应民意。把依法行政作为政府工作的基本准则，严格按照法定权限和程序行使权力、履行职责，自觉接受人大法律监督和工作监督、政协民主监督，高度重视司法监督、新闻舆论监督和人民群众监督，充分听取民主党派、工商联、无党派人士和各人民团体的意见。认真办理人大代表建议、政协提案，努力提高满意度和落实率。规范政府信息公开，提升行政权力网上公开透明运行水平，让权力真正在阳光下运行。办好市长公开电话、市长信箱和行风热线，畅通诉求渠道，及时排忧解难。

　　大力弘扬调研之风、务实之风、清廉之风。以“素质转型”促“服务转型”，以“能力升级”促“效能升级”，扎实推进学习型政府建设。深入开展调查研究，切实增强工作的预见性、针对性和创造性。围绕“十二五”规划，加强宏观政策研究，组织开展专题调研，制定出台具体措施。坚持扑下身子抓落实，勇于担当干事业，挤出更多的时间和精力用于谋划发展、服务基层，推动工作、多办实事。继续深化“三服务”活动，全力做好政策、土地、资金、人才、用工等综合协调服务工作。强化政府公共服务职能，优化财政支出结构，对带动性强、群众呼声高、改善民生快的项目优先安排、加快实施。坚持从严治政，建立健全统一规范的责任追究制度。继续清理依附行政权力的中介收费，加强对重点领域、重点行业、重点部位的监管，加大审计和监察力度，为基层和群众提供公平公正、优质高效的公共服务。加强廉政建设，坚决惩治和预防腐败，保持清正廉洁的良好形象。

　　各位代表，新的征程已经开启，新的使命催人奋进。让我们紧密团结在以胡锦涛同志为总书记的党中央周围，全面落实科学发展观，在中共泰州市委的领导下，依靠和团结全市人民，解放思想，抢抓机遇，锐意进取，奋力拼搏，加快建设更高水平小康社会，为“推进富民强市、建设美好泰州”、早日基本实现现代化而努力奋斗!
